# Antimicrobial Treatment of *Staphylococcus aureus* in Patients With Cystic Fibrosis

**DOI:** 10.3389/fphar.2019.00849

**Published:** 2019-08-07

**Authors:** Susanna Esposito, Guido Pennoni, Valeria Mencarini, Nicola Palladino, Laura Peccini, Nicola Principi

**Affiliations:** ^1^Pediatric Clinic, Cystic Fibrosis Center of Umbria Region, Department of Surgical and Biomedical Sciences, Università degli Studi di Perugia, Perugia, Italy; ^2^Pediatric Unit, Cystic Fibrosis Center of Umbria Region, Branca Hospital, Branca, Italy; ^3^Università degli Studi di Milano, Milan, Italy

**Keywords:** antibiotics, cystic fibrosis, MRSA, pulmonary exacerbation, Staphylococcus aureus

## Abstract

*Staphylococcus aureus* is a ubiquitous human commensal pathogen. It is commonly isolated in cystic fibrosis (CF) patients and is considered one of the main causes of the recurrent acute pulmonary infections and progressive decline in lung function that characterize this inherited life-threatening multisystem disorder. However, the true role of *S. aureus* in CF patients is not completely understood. The main aim of this narrative review is to discuss the present knowledge of the role of *S. aureus* in CF patients. Literature review showed that despite the fact that the availability and use of drugs effective against *S. aureus* have coincided with a significant improvement in the prognosis of lung disease in CF patients, clearly evidencing the importance of *S. aureus* therapy, how to use old and new drugs to obtain the maximal effectiveness has not been precisely defined. The most important problem remains that the high frequency with which *S. aureus* is carried in healthy subjects prevents the differentiation of simple colonization from infection. Moreover, although experts recommend antibiotic administration in CF patients with symptoms and in those with persistent detection of *S. aureus*, the best antibiotic approach has not been defined. All these problems are complicated by the evidence that the most effective antibiotic against methicillin-resistant *S. aureus* (MRSA) cannot be used in patients with CF with the same schedules used in patients without CF. Further studies are needed to solve these problems and to assure CF patients the highest level of care.

## Introduction

*Staphylococcus aureus* is a ubiquitous human commensal pathogen. It is commonly isolated in cystic fibrosis (CF) patients and is considered one of the main causes of the recurrent acute pulmonary infections and progressive decline in lung function that characterize this inherited life-threatening multisystem disorder (Cogen et al., 2015). However, the true role of *S. aureus* in CF patients is not completely understood (Hurley, 2018). Differentiation of *S. aureus* infection (i.e., presence of *S. aureus* in the respiratory tract associated with significant respiratory symptoms) from simple *S. aureus* colonization (i.e., presence of *S. aureus* in the airways without any clinical manifestation) in CF patients is frequently impossible.

Contrary to what has been demonstrated for *Pseudomonas aeruginosa* (Lund-Palau et al., 2016), it is not definitively known whether prevention of *S. aureus* colonization is an effective and safe measure to reduce the risk of early lung infections (Smyth and Rosenfeld, 2017). Moreover, although there is general agreement that antibiotics must be administered to treat acute *S. aureus* infections (Akil and Muhlebach, 2018), it is not known which kind of therapy is the most effective to reduce the risk of chronic *S. aureus* disease, particularly when methicillin-resistant *S. aureus* (MRSA) strains are the causative agents (Muhlebach, 2017). Finally, when persistence of *S. aureus* in the respiratory tract occurs, there is no agreement on how best to eradicate *S. aureus* from the lung (Ahmed and Mukherjee, 2018). The main aim of this narrative review is to discuss the present knowledge of the role of *S. aureus* in CF patients.

## Carriage of *Staphylococcus aureus* in Healthy Subjects and in CF Patients

*S. aureus* colonization is common in healthy subjects. Colonization occurs in the first days of life, as more than 70% of neonates have basal cultures positive for this pathogen. Moreover, approximately 45% of infants become persistently colonized during the first 8 weeks after birth (Peacock et al., 2003). *S. aureus* carriage declines with increasing age but remains significant even in adolescents and adults, among whom 30% or more are found to be carriers (Kenner et al., 2003; Anwar et al., 2004; Bischoff et al., 2004; Leman et al., 2004). The frequency of carriage detection is strictly related to the method used to collect respiratory secretions. Traditionally, only nasal swabs are used, but it has been shown that a nonmarginal number of subjects carry *S. aureus* in the oropharynx. Consequently, a higher prevalence of carriage is shown when both nasal and pharyngeal swabs are simultaneously collected. Esposito et al. (Esposito et al., 2014) studied 497 healthy subjects aged 6–17 years and found that 264 (53.1%) were *S. aureus* carriers: 129 (25.9%) oropharyngeal carriers and 195 (39.2%) nasal carriers, of whom 60 (12.1%) were both oropharyngeal and nasal carriers. All these findings suggest that the likeliness of a nasal or oropharyngeal swab being positive for *S. aureus* accurately reflecting the etiology of a lower respiratory tract infection is poor and that the use of nasal/oropharyngeal swabs to identify *S. aureus* lung infections can lead to incorrect results with significant overestimation.

Differentiation of simple carriers from truly infected patients seems, however, even more difficult in CF patients, as the frequency of carriage in these subjects has been found to be greater than in individuals without CF. A comparison of swab and bronchoalveolar lavage *S. aureus* cultures carried out in CF children <5 years of age showed that the positive predictive value of swabs was only 64% (Rosenfeld et al., 1999). Some factors could explain the higher tendency of CF patients to become *S. aureus* carriers. CF infants have an earlier presence of *S. aureus* in the nasopharynx than healthy controls probably because they have a defense defect against bacteria that can favor respiratory tract colonization (Prevaes et al., 2016). The evidence that CF pigs, when infected by bacterial pathogens, develop lung disease and exhibit defective bacterial eradication at birth strongly supports this hypothesis (Stoltz et al., 2010). Moreover, unlike other bacteria, *S. aureus* can grow very well in anaerobiosis, a condition compatible with the CF ecosystem where reduced mucociliary transport, persistent mucus hypersecretion, and increased height of the luminal mucus layer lead to the development of significant hypoxic conditions in the mucus plugs (Goss and Muhlebach, 2011). However, contrary to what occurs in healthy subjects, who in most cases, although being carriers, remain asymptomatic (Wertheim et al., 2005), in CF patients, early and persistent *S. aureus* colonization, particularly when small colony variants (SCVs) are detected, may be associated with a significantly worse respiratory outcome (Wolter et al., 2013). Moreover, *de novo* *S. aureus* acquisition at 3 years of age was associated with the development of more bronchiectasis and lower FEF25–75% predicted at 5–7 years (Caudri et al., 2018).

The risk that colonization with *S. aureus* could lead to the development of infections with rapid structural and functional deterioration of the lower respiratory tract explains why prophylaxis of *S. aureus* colonization has been considered a potential measure to protect children with CF from the first months of life.

## Prophylaxis of *Staphylococcus aureus* Colonization

Anti-staphylococcal prophylaxis with a narrow spectrum antibiotic, such as flucloxacillin, is recommended in the UK from diagnosis in the neonatal period to the end of the third year of life with possible extension up to 6 years of age (National Institute for Health and Care Excellence, 2017). This recommendation is supported by the results of some studies carried out several years ago. Cefalexin prophylaxis for 2 years in the ambulatory management of CF patients colonized by *S. aureus* was shown to be associated with a significant reduction in the number of respiratory illnesses, hospitalizations for respiratory problems, and use of antibiotics compared to patients receiving placebo (Loening-Baucke et al., 1979). Moreover, for 24 months, Weaver et al. (Weaver et al., 1994) monitored a group of children with CF treated from the first weeks of life with continuous oral flucloxacillin or with episodic antimicrobials as clinically indicated. They found that children who were not given prophylaxis had more frequent cough and a greater number of *S. aureus* isolates in their sputum than infants on prophylaxis. Moreover, children with CF were more frequently hospitalized and had longer stays in the hospital than flucloxacillin-treated subjects.

However, more recent studies have raised doubts both about the efficacy and about the safety of prophylaxis. Prophylaxis-related problems are highlighted by the conclusions of a Cochrane review in which four studies published up to September 2016 were analyzed (Smyth and Rosenfeld, 2017). Prophylaxis commenced early in infancy and continued up to 6 years of age did not cause significant adverse events and reduced the number of children with one or more isolates of *S. aureus* during the study period. However, continuous antibiotic administration did not lead to any true clinical advantage. No differences between treated and untreated children were evidenced with respect to lung function, nutrition, hospital admissions, and additional courses of antibiotics. Moreover, it could not be excluded that antibiotic prophylaxis could cause an increased risk of *P. aeruginosa* acquisition. Pooling data from the four studies demonstrated a trend towards a lower cumulative isolation rate of *P. aeruginosa* in the prophylaxis group at 2 and 3 years and a trend towards a higher rate from 4 to 6 years.

Furthermore, the limit of the lack of information on the true efficacy and safety of *S. aureus* prophylaxis was not resolved by the results of a number of studies not included in the previously cited meta-analysis. The results were clearly conflicting. The administration of amoxicillin–clavulanate was found to be ineffective in both *S. aureus* and *P. aeruginosa* acquisition (Douglas et al., 2009). Long-term use of anti-staphylococcal drugs, mainly oral cephalosporins, reduced *S. aureus* colonization but was associated with an increased risk *of P. aeruginosa* acquisition (Ratjen et al., 2001). Finally, the most disconcerting finding on the use of *S. aureus* prophylaxis comes from the results of a study in which data collected in the UK, where prophylaxis was recommended, were compared to data collected in the USA, where antibiotics were given only intermittently as required (Hurley et al., 2018). A total of 1,074 UK and 3,677 US children with CF were recruited from birth or from their first evaluation and followed until the age of 4 years. Earlier acquisition of both *S. aureus* and *P. aeruginosa* was evidenced in US children, suggesting a positive effect of prophylaxis. However, when the results collected in the UK were separately analyzed, it was shown that children who received flucloxacillin had an increased risk of acquiring *P. aeruginosa* (hazard risk [HR], 2.53; 95% confidence interval [CI], 1.71–3.74; *p* < 0.001] and no advantage regarding *S. aureus* acquisition (HR 1.22; 95% CI, 0.74–2.0; *p* = 0.43).

However, as the efficacy and safety of antibiotic prophylaxis remains debated, the Cystic Fibrosis Foundation of the USA does not recommend the prescription of anti-*S. aureus* agents for prophylaxis (Cystic Fibrosis Foundation, 2013).

## *Staphylococcus aureus* Infection in CF Patients

In the first decade of life, *S. aureus* is the most common pathogen identified in the airways of CF patients with respiratory symptoms. Only later does *P. aeruginosa* become prevalent, although *S. aureus* still plays a relevant role as a cause of exacerbations [Cystic Fibrosis Foundation (US), 2017]. However, in recent years, the prevalence of *S. aureus* infection has increased, as evidenced by Razvi et al. (Razvi et al., 2009), who analyzed the Cystic Fibrosis Foundation Patient Registry of the USA from 1995 to 2005 and calculated that, during this period, the prevalence of both MSSA and MRSA infections progressively increased (MSSA infection rate of 21.7% in 1995 and 33.2% in 2005; MRSA infection rate of 0.1% in 1995 and 17.2% in 2005).

Due to the production of several virulence factors (Akil and Muhlebach, 2018), the presence of *S. aureus* in the respiratory tract is associated with significant lung alterations. The development of a relevant inflammatory response, which is more severe than the inflammatory response to other common respiratory bacteria and less severe than only the response to *P. aeruginosa* (Gangell et al., 2011), further increases damage to the lung structure and functions. In the bronchoalveolar lavage fluid of CF children with an *S. aureus* infection, cell counts are increased as are the levels of interleukin-8, neutrophil enzymes (myeloperoxidase and neutrophil elastase), and markers of oxidative stress (Khan et al., 1995; Rosenfeld et al., 2001; Brennan et al., 2005; Sly et al., 2009). Although with some exceptions (Thomas et al., 1998; Miall et al., 2001; Sawicki et al., 2008), studies have shown that lung damage is greater, and death occurs more frequently and earlier when MRSA strains are the cause of the infection. Ren et al. (Ren et al., 2007) evaluated forced respiratory volume in 1 s (FEV1) of 1834 CF patients positive for *S. aureus* alone, according to the presence of MRSA or MSSA in their respiratory secretions. In all the subjects, regardless of age, FEV1 was significantly lower in patients with MRSA than in those with MSSA (*p* < 0.001). Moreover, the likelihood of hospitalization and treatment with antibiotics was significantly increased in patients with MRSA compared to those with MSSA. However, lung function declines at a faster rate when MRSA infection persists rather than in cases of incident detection. Over a 2-year period, Sawicki et al. (Sawicki et al., 2008) found no change in the predicted rate of FEV1% decline in patients with incidental MRSA detection. In contrast, Dasenbrook et al. (Dasenbrook et al., 2008), who studied 1,732 individuals aged 8–21 years with persistent MRSA infection (≥3 MRSA cultures), followed for an average of 3.5 years, reported that the average FEV1 decline in these patients was 43% more rapid than in those without MRSA (difference, −0.62% predicted/year; 95% CI, −0.70 to −0.54; *p* < 0.001).

The impact of MRSA on mortality is highlighted by the study carried out by Dasenbrook et al. (Dasenbrook et al., 2010). These authors retrospectively analyzed medical charts of 19,833 CF patients aged 6 to 45 years followed between January 1996 and December 2006 in the USA to compare survival between CF patients with and without respiratory tract MRSA. The mortality rate was 18.3 deaths (95% CI, 17.5–19.1) per 1,000 patient-years in patients without MRSA and 27.7 deaths (95% CI, 25.3–30.4) per 1,000 patient-years in those with MRSA. After adjustment for time-varying covariates associated with severity of illness, MRSA was found to be associated with a higher risk of death (95% CI, 1.11–1.45). On the other hand, the importance of MRSA as a cause of death seems confirmed by the evidence that when MRSA is eradicated from the respiratory tract, the risk of death returns to similar levels detected in MSSA-positive patients.

All these findings explain why the eradication of *S. aureus* from the respiratory tract of CF patients is presently considered essential to limit the negative evolution of the lung structure and functions. However, to reach this goal, it seems mandatory not only to prescribe effective antibiotic treatment but also to intervene, when possible, in the factors that favor persistence of *S. aureus* and the development of chronic infection.

## Factors Associated With the Persistence of *Staphylococcus aureus* in CF Patients

Several risk factors for persistent *S. aureus* infection in people with CF have been identified. The persistence of MRSA has been associated with receiving care at a CF center with increased MRSA prevalence, the presence of pancreatic insufficiency, CF-related diabetes, and the number of hospitalizations per year (Akil and Muhlebach, 2018). However, the most important reason seems to be the ability of the pathogen to develop adaptive mechanisms that allow it to resist antibiotic pressure and host defenses. Transformation into SCVs, growth under anaerobic conditions, biofilm formation, and development of persister isolates have been described. Although there are differences according to the substrate used for culture or the ability to grow under CO_2_ (Gomez-Gonzalez et al., 2010), *S. aureus* SCVs are generally characterized by mutations in metabolic genes that cause growth deficiency and depressed but not excluded α-cytotoxin activity. This allows the pathogen to viably persist inside host cells. However, when lysis of the host cell occurs, intracellular *S. aureus* once again become extracellular and can invade the adjacent tissue. This explains why SCVs can often be retrieved in patients suffering from recurrent and therapy-refractory infections. Finally, as SCVs appear as small, smooth colonies with slow growth on a culture plate, they are difﬁcult to detect and frequently overlooked or misidentiﬁed (Kahl et al., 2016).

However, when correct culture conditions are used, SCVs can be detected in a relevant number of CF patients. In a 34-month prospective study, it was found that SCVs could be identified, alone or in combination with normal *S. aureus*, in one-third of the cases and that in most of the patients, these variants remained in the respiratory tract for a long time, up to 31 months (Kahl et al., 1998). The presence of SCVs is associated with persistent infection, worse lung function, resistance to several antibiotics, and coinfection with *P. aeruginosa* (Besier et al., 2007; Schneider et al., 2008; Wolter et al., 2013).

Regarding sensitivity to antibiotics, most SCVs are MRSA. In a recent study (Suwantarat et al., 2018) carried out in the USA from July to December 2014 in which 483 *S. aureus*-positive respiratory samples were evaluated, it was shown that individuals with SCV MRSA more frequently (93%) had persistent MRSA infection (≥4 MRSA-positive respiratory cultures in the previous 18 months) than those with non-SCV-MRSA (39%, *p* < 0.001). Moreover, respiratory function in patients with MRSA SCVs was worse than in those with non-SCV-MRSA, as evidenced by a statistically significant lower mean predicted FEV1% (*p* < 0.001), and the use of trimethoprim/sulfamethoxazole (TMP/SMX) and tetracyclines was higher (*p* < 0.001 and *p* < 0.004, respectively). Finally, although 100% of SCVs were susceptible to vancomycin and ceftaroline, most of them were highly susceptible to linezolid (86%), rifampin (86%), and tetracycline (86%), and susceptibility to clindamycin, TMP/SMX, erythromycin, and moxifloxacin (4%) was poor (18%, 18%, 4%, and 4%, respectively). Moreover, a nonmarginal number of isolates had a vancomycin minimum inhibitory concentration (MIC) of 2 µg/dl, which is at the upper end of the susceptibility range (Chen et al., 2014).

The emergence of SCVs depends on several factors, including previous exposure to antibiotics and coinfection with *P. aeruginosa*. Besier et al. (Besier et al., 2007) reported that previous treatment with TMP/SMX was an independent risk factor for the detection of SCVs. Similar findings were reported by Schneider et al. (Schneider et al., 2008), who showed that carriers of *S. aureus* SCVs were pretreated more often and for longer periods with systemic aminoglycosides (*p* = 0.02) and TMP/SMX (*p* = 0.001).

*S. aureus/P. aeruginosa* coinfection is relatively common in CF patients. Several studies have shown that SCVs are detected more frequently in cases of coinfection than when only *S. aureus* is identified in respiratory secretions (Kahl et al., 1998; Besier et al., 2007; Schneider et al., 2008). This finding is considered the consequence of a survival strategy used by *S. aureus* to overcome the negative effects of several exoproducts secreted by *P. aeruginosa* (Hotterbeekx et al., 2017). *In vitro* studies have shown that molecules such as 4-hydroxy-2-heptylquinoline *N*-oxide (HQNO) (Hoffman et al., 2006) and pyocyanin (Tashiro et al., 2013) produced by *P. aeruginosa* inhibit the cytochrome system, hindering oxidative respiration and *S. aureus* growth and favoring the formation of SCVs (Filkins et al., 2015). This finding clearly highlights the importance of effective prophylaxis and the treatment of *P. aeruginosa* colonization and infection to reduce the risk of SCV formation and highlights the greater problems in treating *S. aureus*. After SCV induction, further metabolic changes in *S. aureus* occur. These changes promote the intracellular persistence of *S. aureus* (Atalla et al., 2011) and its tendency to form biofilms (Mitchell et al., 2010). As most of the drugs used to treat bacterial infections in CF patients remain in the extracellular space and do not have significant intracellular bactericidal activity, intracellular SCVs are not killed, and chronic infection is established. Moreover, SCVs remain potentially able to cause acute infection, particularly when cells are dispersed from the biofilm, which can result in the spread of pathogens (Moormeier and Bayles, 2017; Akil and Muhlebach, 2018).

The formation of biofilms further contributes to the persistence of infections and the risk of acute exacerbation because the encasement of bacteria in a polymer-based matrix reduces the efficacy of host defenses and antibiotic activity. Antibodies and macrophages poorly penetrate the biofilm structure, and pathogens incorporated into biofilms have reduced susceptibility. Moreover, they survive high concentrations of antibiotics although the tested MIC does not change (Waters et al., 2016).

The development of persister cells is the last factor that can explain chronic *S. aureus* infection and recurrent acute exacerbations in CF patients. Persistence is the ability of a pathogen to tolerate lethal doses of antibiotics despite undergoing no genetic changes (Brauner et al., 2016). Persistent cells are dormant or phenotypic variants that are commonly present in the general population of a given bacterial pathogen and can be progressively selected as a consequence of repeated antibiotic treatments that reduce normal strains and favor the emergence of persisters. In CF patients, this has been clearly evidenced for *P. aeruginosa* (Smith et al., 2006), and it is suggested to occur even for *S. aureus.*

## Treatment of Acute and Chronic *Staphylococcus aureus* Infection in CF Patients

CF patients with *S. aureus* can be divided into four different groups: those with the first or an early *S. aureus* infection that can be asymptomatic or symptomatic and those with chronic infections that can be in stable clinical conditions or with an acute respiratory exacerbation.

Treatment of CF patients with their first or an early *S. aureus* infection without symptoms is debated because, as previously reported, it is practically impossible to differentiate between simple colonization and asymptomatic infection. Moreover, unneeded antibiotic administration can be followed by several problems, including the emergence of antibiotic resistance and *P. aeruginosa* colonization.

The use of antibiotics in symptomatic patients is, on the contrary, recommended as well as the treatment of stable subjects with chronic infection, although it is not definitively established whether treatment can eradicate *S. aureus* and modify the clinical course of CF. Several attempts to clarify these problems have been made, but the results do not permit firm conclusions to be drawn because most of the studies were noncontrolled case series, enrolled very few patients, and used different definitions of *S. aureus* eradication and treatment schedules.

Until the first years of the 1990s, when MRSA was uncommon, attempts to treat and eradicate *S. aureus* were mainly based on a combination of two antibiotics from a choice of a semi-synthetic β-lactamase-resistant drug (flucloxacillin or dicloxacillin), rifampicin and fusidic acid. An antibiotic course of 2–4 weeks was considered effective in most of the patients. In nonresponding subjects, a second course was recommended (Döring and Hoiby, 2004). A retrospective study carried out in Denmark involving 191 CF patients and 2,349 antibiotic courses from 1965 to 1979 showed that *S. aureus* could be persistently eradicated from sputum in 74% of the cases. A second antibiotic course lasting 2 or 4 weeks was successful in most of the remaining patients, as evidenced by the persistence of infection for 6 months in only 9% of the cases (Szaff and Høiby, 1982).

The approach to *S. aureus* infection has become significantly more complicated with the emergence of MRSA. Although MRSA has been identified worldwide, the frequency of detection is particularly high in Asia, Malta, and North and South America (Stefani et al., 2012), where the distribution of this pathogen is rapidly increasing. In the USA, for example, the prevalence in 2017 was 25.9%, compared to 2% in 1999 [Cystic Fibrosis Foundation (US), 2017]. An increase in MRSA detection was also recently evidenced in Europe, where it was no higher than 3% in most countries until a few years ago (Goss and Muhlebach, 2011). In contrast, in 2016, the European Centre for Disease Control and Prevention reported that the percentage of MRSA among all *S. aureus* isolates was above 25% in 7 of the 29 reporting countries from the European Union or European Economic Area (European Centre for Disease Prevention and Control, 2018).

Although a number of patients with their first or early MRSA-positive cultures can clear the pathogens without therapy because they are simply colonized, several studies have evaluated the impact of antibiotics in these subjects. Oral and parenteral antibiotics, sometimes associated with inhaled drugs such as vancomycin (Campbell et al., 2016), have been used; in most cases, a certain positive effect was evidenced (Garske et al., 2004; Macfarlane et al., 2007; Vanderhelst et al., 2013; Kappler et al., 2016; Muhlebach et al., 2017; Dolce et al., 2019). A few examples can illustrate which protocols were used and which results were obtained. In newly colonized patients aged 0.6–39.6 years, an open prospective study carried out in Germany between January 2002 and December 2012 (Kappler et al., 2016) showed that long‐term eradication, rated by the microbiological status in the third year after first detection, could be obtained in 84% of the cases (31/37 patients) with a three-step protocol. Initially, patients were treated over 3 weeks with two intravenous (IV) antibiotics, chosen according to pathogen sensitivity. Hygienic measures and topical therapy for 5 days were also used. This first treatment step was followed by a 6‐week period with dual oral antibiotic therapy and inhalation of vancomycin. Finally, each new detection of MRSA was treated with 6 weeks of inhalation of vancomycin and topical therapy for 5 days. In a randomized controlled trial carried out between April 2011 and September 2014 in the USA (Muhlebach et al., 2017) in patients aged 4–45 years with their first or early (≤2 positive cultures within 3 years) *S. aureus* infection, eradication was defined as a negative MRSA respiratory culture 28 days after randomization, and treatment was based on oral TMP/SMX or, if the patient was sulfa-allergic, minocycline plus oral rifampin, chlorhexidine mouthwash for 2 weeks, nasal mupirocin and chlorhexidine body wipes for 5 days, and environmental decontamination for 21 days. Eradication was observed in 82% of cases receiving antibiotics and in 26% of controls. On day 84, 55% of treated subjects and only 10% of controls remained MRSA-negative. In an Italian randomized controlled trial performed between July 2013 and April 2016 (Dolce et al., 2019), a significantly longer period without MRSA in respiratory secretions (three negative cultures in 6 months) was considered indicative of eradication. Moreover, although the same antibiotics were used as prescribed in the USA trial, treatment was given for a longer period (21 days). Moreover, skin and surface decontamination was not added. A lower level of MRSA positivity was demonstrated in treated patients, although the differences compared to controls did not reach statistical significance. However, despite some positive results, a recent Cochrane review of published studies (Lo et al., 2018) concluded that presently there is not enough evidence to support the use of the treatment schedules suggested in these studies. On the other hand, suggested protocols have not been adequately evaluated in terms of the impact on lung function, mortality, and cost of care. Moreover, some protocols are very complicated and require full compliance by the patient and, in the case of children, by his or her family.

Very few data have been collected on the impact of antibiotic treatment in CF patients with persistent MRSA, and the available data seem to indicate that eradication in these subjects is very difficult. In a recent study carried out in the USA in 2017 (Dezube et al., 2018), in which 29 subjects aged ≥18 years with chronic MRSA infection were enrolled, treatment was based on TMP/SMX or, if intolerant, doxycycline, in association with rifampin for 28 days. Topical decontamination and environmental cleaning were added. Moreover, all the enrolled patients were randomized to receive either inhaled vancomycin or a nebulized placebo. The results were unsatisfactory because treatment was globally poorly effective and inhaled vancomycin did not increase the eradication rate. Only 20% of cases in both groups had an MRSA-negative sputum culture at the end of treatment and 3 months later.

In patients with chronic MRSA infection suffering from an exacerbation, treatment varied according to the severity of disease. An anonymous cross-sectional survey of CF Foundation-accredited care programs in the USA (Zobell et al., 2015) revealed that outpatient treatment in 2013 was based on oral drugs. TMP/SMX was the most commonly prescribed antibiotic in both children (38%) and adults (34%). A combination with rifampin was used in 10% of the cases. For inpatient treatment, linezolid (both IV and oral) and IV vancomycin were the most commonly prescribed drugs. These drugs are considered the drugs of choice to treat exacerbations of *S. aureus* infections by most experts (Mandell et al., 2007; Torres et al., 2009).

## Concerns Regarding the Antibiotics Used to Treat *Staphylococcus aureus* Infections in CF Patients

The use of antibiotics in CF patients deserves particular attention because, in these subjects, liver metabolism of drugs is increased, as is renal clearance. This state means that in many cases, drug dosage has to be increased and serum levels have to be monitored to avoid the risk of toxicity. For example, it is recommended that the dose of β-lactams be increased by 20% to 30% and plasma concentrations of aminoglycosides be monitored (Rey et al., 1998). Moreover, it is common that CF patients simultaneously receive several antibiotics to treat *S. aureus* and *P. aeruginosa* that frequently coinfect these subjects. The use of multiple therapies increases the risk of antibiotic-related adverse events and negative interference between drugs. Finally, some of the antibiotics commonly used in CF patients have limitations that must be considered to assure an effective therapeutic outcome.

One of the best examples in this regard is provided by vancomycin. This drug has poor penetration in both the lung and the biofilm (Cruciani et al., 1996), which can cause problems with MRSA eradication, especially when the pathogen has an MIC in the upper range of sensitivity (van Hal et al., 2012). However, the recommended vancomycin dose is 15 mg/kg every 8 h because it has been demonstrated that, in adults, this dosage generally assures the achievement of the pharmacokinetic parameters [a ratio of the 24-h area under the concentration–time curve (AUC) to the bacterial MIC ≥400 and a trough concentration of 15–20 μg/ml] that have been associated with elimination of sensitive MRSA (Liu et al., 2011).

However, although the pharmacokinetics of vancomycin in adults with CF is similar to that of healthy adults (Pleasants et al., 1996), the same is not true in children. In children with CF, vancomycin pharmacokinetics differs, and the recommended dosage does not always assure the maximal drug efficacy. A mean dose of 17.4 ± 4.4 mg/kg resulted in a mean serum trough concentration of only 10.1 ± 3.8 mg/L and a mean daily AUC of only 282.5 ± 816.9 mg (Stockmann et al., 2013). This result means that, in children, higher doses or continuous monitoring of drug concentration is necessary to verify the achievement of effective trough levels. In contrast, the use of high doses alone requires monitoring because vancomycin is nephrotoxic and higher than recommended trough serum levels have been associated with significant renal insufficiency (Carreno et al., 2014). The association with other nephrotoxic drugs, such as aminoglycosides, further indicates the need for monitoring. To reduce the risk of treatment failure or toxicity, continuous infusion has been suggested. Children with acute exacerbation that did not achieve adequate trough levels despite being given 15–19 mg/kg every 6 h were successfully treated with continuous infusion of dosages between 31 and 50 mg/kg/day without any significant adverse events (Fung, 2012).

Compared to vancomycin, linezolid has the advantage of being administered not only by IV but also by mouth. However, the dosage required to treat MRSA infections in CF patients has not been definitively established. In adults, administration of 600 mg twice a day is considered effective to eradicate MRSA with an MIC of ≤1 µg/ml, but an addition of a third daily dose seems necessary when the MIC is ≥2 µg/ml (Keel et al., 2011). In children with CF, no effective dosage has been defined. In a study in which the approved dose of 10 mg/kg three times a day was used, it was found that no subject achieved the target AUC/MIC ≥83 and that the younger patients had the lowest AUC/MIC values (Santos et al., 2009). These findings seem to indicate that doses higher than recommended are needed to treat MRSA infections in pediatric patients, particularly in younger children. Unfortunately, this suggestion collides with the evidence that higher linezolid doses are associated with nonmarginal gastrointestinal and hematological adverse events (Chiappini et al., 2010).

Some concerns have also been raised for other drugs commonly used to treat *S. aureus* infections. Tetracyclines, TMP/SMX, and fusidic acid generally show low levels of resistance *in vitro* (Champion et al., 2014) and have been largely used in the attempt to eradicate MSSA and MRSA (Muhlebach et al., 2017; Dolce et al., 2019). However, tetracyclines cannot be used in children younger than 8 years old because of the risk of developing adverse events (Smith et al., 2001). TMP/SMX seems to have different pharmacokinetic characteristics in CF patients, with a shorter half-life and an increased total body clearance time (Reed et al., 1984), which suggests the need for an increase in daily dosage, but doses adequate to obtain the maximal effect have not been defined. Moreover, data regarding the efficacy of TMP/SMX in MRSA exacerbations are lacking. Among the novel antibiotics effective against MRSA, ceftaroline has been used to treat MRSA infections in CF patients. In a study of the pharmacokinetic characteristics of ceftaroline in children and young adults with CF, it was found that, compared to healthy individuals, ceftaroline had a significantly lower half-life and required higher than recommended doses to achieve blood concentrations >60% of the MRSA MIC in all the patients (Le et al., 2017; Barsky et al., 2018). Some studies have reported that ceftaroline can be effective in the treatment of MRSA infections in non-CF patients. The mean clinical cure rate was 74% across 379 patients (Cosimi et al., 2017). However, experience in CF is extremely limited (Cannavino et al., 2016), and no information is available regarding safety and tolerability when higher doses are administered. Clindamycin cannot be recommended in CF patients as it has been shown that, in patients with CF, prevalence of inducible clindamycin resistance is significantly higher than that in non-CF subjects (48% vs 8%; *p* < 0.01) (Moore et al., 2008). Finally, although novel lipoglycopeptide drugs oritavancin (Stewart et al., 2017) and dalbavancin (Bouza et al., 2018) are effective *in vitro* against multidrug-resistant *S. aureus*, they are not licensed for use in children and cannot be presently recommended to treat CF patients as presently there is little evidence for their use in lung infection.

## Conclusions

Despite the fact that the availability and use of drugs effective against *S. aureus* have coincided with a significant improvement in the prognosis of lung disease in CF patients, clearly evidencing the importance of *S. aureus* therapy, how to use old and new drugs to obtain the maximal effectiveness has not been precisely defined. The most important problem remains that the high frequency with which *S. aureus* is carried in healthy subjects prevents the differentiation of simple colonization from infection. This problem is particularly relevant when detection of *S. aureus* occurs in children without signs and symptoms of disease and explains why there is no consensus on the prophylactic use of antibiotics.

Moreover, although experts recommend antibiotics in CF patients with symptoms and in those with persistent detection of *S. aureus*, the best antibiotic approach has not been established. It is not precisely defined which are the drugs of choice to treat MRSA infections. Furthermore, more information is needed as far as the dosage and duration of antibiotic administration are concerned. Finally, the best way to definitively evaluate effectiveness of antibiotic therapy has to be defined in order to assure CF patients the highest level of care.

## Author Contributions

SE proposed the project and wrote the first draft of the manuscript; GP, VM, and NPa critically revised the text and gave a substantial scientific contribution; LP performed the literature analysis; NPr co-wrote the manuscript and supervised the project. All of the authors approved the final version of the manuscript.

## Funding

This study was supported by a grant from Fondazione Cassa di Risparmio di Perugia (#3368-2018).

## Conflict of Interest Statement

The authors declare that the research was conducted in the absence of any commercial or financial relationships that could be construed as a potential conflict of interest.
